# A Functional Model of Kitsch and Art: Linking Aesthetic Appreciation to the Dynamics of Social Motivation

**DOI:** 10.3389/fpsyg.2018.02437

**Published:** 2019-01-28

**Authors:** Stefan A. Ortlieb, Claus-Christian Carbon

**Affiliations:** ^1^Department of General Psychology and Methodology, University of Bamberg, Bamberg, Germany; ^2^Bamberg Graduate School of Affective and Cognitive Sciences (BaGrACS), Bamberg, Germany; ^3^Forschungsgruppe EPÆG (Ergonomics, Psychological Æsthetics, Gestalt), Bamberg, Germany

**Keywords:** empirical aesthetics, dynamics of appreciation, art perception, kitsch, autonomy, security, arousal, Zurich Model of Social Motivation

## Abstract

With the advent of modernity, change and novelty have become the core values of artistic production. At the same time the derogatory term “kitsch” was coined to contrast truly ground-breaking artistic achievements. In this article, we argue that kitsch and avant-garde art ideally represent two complementary types of aesthetic experience: a fluent one that allows for immediate emotional gratification (kitsch) and a disfluent one that requires cognitive elaboration (art). We make a case that preferences for the one or the other are dynamically related to a set of conflicting needs which constitute the basic dilemma of human attachment behavior: needs for safety and intimacy versus needs for arousal and autonomy. Based on the *Zurich Model of Social Motivation* we hypothesize that social distance regulation and aesthetic liking are synchronized via notions of self-efficacy and autonomy: Whenever we feel safe and self-sufficient, an appetence for arousal (curiosity) is likely to arise that increases our interest in unfamiliar conspecifics as well as in innovative, cognitively challenging aesthetic stimuli (art). By contrast, when we feel vulnerable and dependent, a longing for safety and relatedness (nostalgia) attracts us not only to familiar and trustworthy individuals but also to conventional aesthetic stimuli charged with positive emotions (kitsch). This theoretical framework offers an integrative perspective on dynamics of aesthetic liking in that it unites a wide variety of phenomena from anthropology, developmental, and cognitive psychology with concepts and findings from art history, sociology of art, and empirical aesthetics.

## Introduction

In aesthetics, there is nothing more persistent than change (Martindale, 1990; Carbon, 2011). Art historians have defined different epochs to account for major changes in style and content on a cultural level. For centuries, such discontinuities in the arts as well as in fashion have coincided with fundamental changes in society: A new political order seems to call for new aesthetic conventions.^1^ With the onset of modernity, however, innovation itself has become the touchstone of artistic production in the Western world obliging artists to “criticism of tradition” (Cǎlinescu, 1987, p. 3). Artists who aspire a particularly advanced position in this rebellion against well-tried aesthetic conventions are identified with a term from French military jargon (Cǎlinescu, 1987): the *avant-garde*. The derogatory label *kitsch* has emerged along with the idea of avant-garde art^2^ (Greenberg, 1939). In art criticism, it has been used to contrast unique and progressive artistic achievements with the outdated, overly simplistic and consoling commodities of popular culture (Greenberg, 1939; Simon-Schäfer, 1980; Kulka, 1996).

On an individual level, similar changes in aesthetic liking can be observed in the context of intergenerational conflict: Puberty is not only a stage of profound physical transformation, but also of social reorientation and aesthetic reevaluation. As a young person’s claim for autonomy awakens, a shift in aesthetic preferences takes place: For instance, choice of music, clothing, and hairstyle are no longer aligned with their parents’ aesthetic standards. Quite the contrary, as curiosity for the exciting outside world—especially peers and idols—intensifies, the familiar sphere of the parental home becomes increasingly “dull,” “stuffy,” and “kitschy” (Stemmle, 1931; Dettmar and Küpper, 2007). Avenarius (1920) reported a similar conflict among artists of the early 20th century when a young generation of artists tried to distinguish themselves from their well-established predecessors by scorning them as “Kitschiers” (p. 222). Again, the term kitsch appears as a symptom of conflict between tradition and innovation.

Recent findings from social psychology and empirical aesthetics provide additional indication for a dynamic interrelation of aesthetic appreciation and social motivation on a situational level: Landau et al. (2006), for instance, observed that mortality concerns diminished liking of Modern art among individuals with a high personal need for structure. In two studies using the mortality salience paradigm there was preliminary evidence for a complementary effect regarding kitsch: Decorative everyday objects were perceived as less kitschy after *in sensu* exposure to existential threats (Raab et al., 2015; Ortlieb et al., 2016a).

By looking at these dynamic phenomena on a cultural, an individual, and a situational level, it seems that the term kitsch tends to resurface in the context of social conflict between the old and the new, and that “[n]o matter how we scorn it, kitsch is an integral part of the human condition” (Kundera, 1984/1999, p. 256). The aim of this paper is to present a functional model that accounts for these dynamics by mapping aesthetic preference for novelty (or familiarity), complexity (or simplicity), and ambiguity (or determinacy), to universal human needs for autonomy, security^3^, and arousal. Before we devise our model, we briefly touch upon three important questions regarding kitsch (and its relation to art):

(1) What do we mean by kitsch?
(2) Why is kitsch aesthetically pleasing?
(3) If it is pleasurable, why is kitsch considered worthless?

These preliminary considerations will set the stage for a model linking appreciation of kitsch and art to the dynamics of social distance regulation (Bischof, 1975, 1993, 2001) and regulatory focus^4^ (Higgins, 1998). In a stepwise approach, each variable of our model is introduced separately before we elaborate on their dynamic interplay. Finally, implications and limitations of the model are discussed with regard to basic (e.g., art perception) and applied research (e.g., product design) on dynamics of aesthetic liking.

## What Do We Mean by Kitsch?

The word “kitsch” and the corresponding aesthetic concept have emerged quite recently (Cǎlinescu, 1987). With some certainty, it can be traced back to the late 1860s when it came into use among artists and art dealers from Munich as a derogatory label for “cheap artistic stuff” (p. 234). To the present day, the origins of the word kitsch remain unclear and have inspired numerous etymological theories (Kluge and Seebold, 2011).^5^ Best (1985) claimed that it derives from Swabian dialect where the verb “Kitschen” originally referred to petty trading, while the noun “Kitsch” was used to designate crude wooden objects, scrap wood, or flotsam. For two reasons, this etymological theory seems rather compelling: Firstly, it allocates the origins of the word kitsch to a local dialect from southern Germany which is where it first came into use in its modern sense. Secondly, it accounts for two socio-economic developments of the 19th century that have prepared the ground for kitsch as a mass phenomenon: According to Greenberg (1939), kitsch is a product of industrialization and universal literacy. With increasing literacy a new market for expendable literature emerged. This demand for affordable reading material was met mainly by haberdashers roaming the land with crates full of mass-produced paperbacks. Furthermore, the indignant reactions of contemporary writers and literature critics to this “reading frenzy” anticipated some of the main tropes of the later kitsch discourse in that pulp literature was scorned as schematic and overly sentimental (Schöberl, 1984; Niehaus, 2002; Dettmar and Küpper, 2007).

Unlike other labels of bad taste, the German word kitsch was adopted by most modern languages (see Ortlieb et al., 2017) and has entered new contexts of use. Meanwhile, it may refer to “virtually anything subject to judgments of taste” (Cǎlinescu, 1987, p. 235): from painting, sculpture, and literature to music, cinema, and TV programs, not to forget architecture, interior decoration, and furnishing. Out of its many contexts of usage, art theory is the most important one (Simon-Schäfer, 1980). The term kitsch has served many authors as a counter-concept to applied art (Pazaurek, 1912/2012), avant-garde art (Greenberg, 1939), or art proper (Simon-Schäfer, 1980). In his essay on *Kitsch and Art* philosopher Tomaš Kulka also dealt with kitsch as a borderline phenomenon of art (Kulka, 1996). Yet, to him the “aesthetic inadequacy” (Cǎlinescu, 1987, p. 236) of kitsch was not self-evident. Instead, he derived three conditions for the application of the kitsch concept from its preferred subjects and stylistic devices in the visual domain. The following kitsch definition is based on these criteria.

**FIGURE 1:**
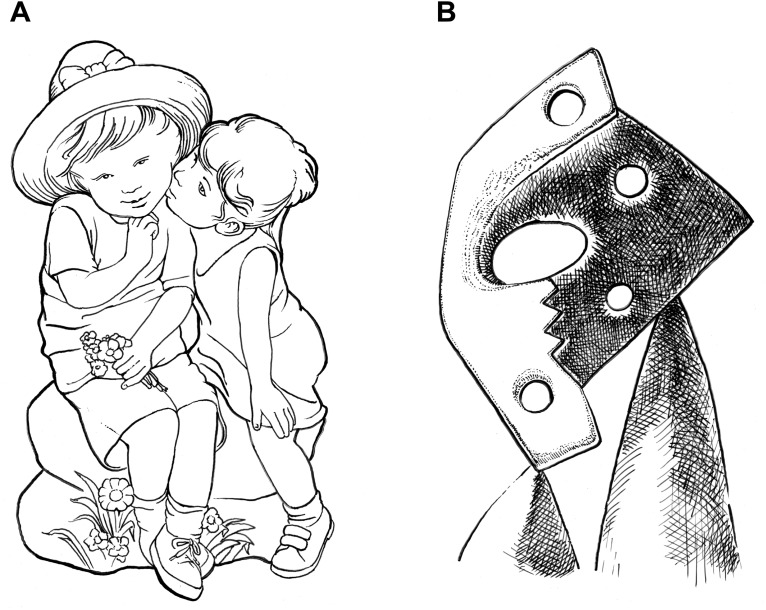
**(A)** *First Kiss* as a typical kitsch subject versus **(B)** *The Kiss* by Picasso (1928). Abstracted drawings by the first author.

According to Kulka (1996), the kitsch concept only applies if the following three conditions are met: First and foremost, kitsch requires content “charged with stock emotions [that] spontaneously triggers an unreflective emotional response” (p. 26). Secondly, this subject matter must be “instantly and effortlessly identifiable” (p. 33), and thirdly, its rendering must not substantially enrich the spectators’ “associations relating to the depicted objects or themes” (p. 37). The first condition suggests that some themes and subjects are more prone to kitsch classification than others. For instance, themes like mothers with babies, turtling doves, or embracing couples are quite typical for kitsch (Kulka, 1996). Universal themes of human existence with a positive emotional valence such as love, birth, childhood, family, or friendship seem particularly evocative of kitsch simply because they represent the “lowest common denominators of experience” (Greenberg, 1939, p. 45). Apart from eliciting feelings of tenderness and affection, they are easily accessible as they are based on common life experience. However, it is important to stress, that kitsch only draws on themes and subjects with a positive emotional valence. For some deeper reason—which we are about to explore in the course of this investigation—it avoids all the unpleasant and troubling aspects of the human condition such as death, illness, loss, and separation. This limitation in terms of emotional valence allows for us to discriminate between an unclouded positive response to kitsch and the multifaceted experience of “being moved” (Menninghaus et al., 2015), that typically involves mixed emotions (Weth et al., 2015) along with indicators for negative affect on a physiological level (Wassiliwizky et al., 2017). Hence, we suggest to modify Kulka’s first precondition as follows: Above all, “kitsch requires a subject matter with a *positive* emotional charge” (Ortlieb and Carbon, 2018, p. 6). The subject *First Kiss* (Figure 1A), for example, seems particularly promising with regard to affective impact since it borrows on several highly emotional themes including first love, friendship, childhood innocence, and, of course, nostalgia. Upon closer examination, Figure 1A also shows how certain stylistic devices are used to increase both likeliness and intensity of a strong emotional response: In order to raise the chance of stimulating personal recollections in the perceiver, the two children are not portrayed as individuals; instead, their features are schematized toward a textbook example of the so-called “baby scheme” (Lorenz, 1943) comprising “a head large in relation to the body, eyes set low in the head, a large protruding forehead, round protruding cheeks, a plump rounded body shape, short thick extremities, soft body surface, and clumsy behavior” (Morreall and Loy, 1989, p. 68). Findings from evolutionary aesthetics illuminate why this scheme of “cuteness” is so closely associated with kitsch: Apart from attracting the beholder’s attention (Brosch et al., 2007), it reliably triggers a positive emotional response that inhibits aggression and promotes caretaking behavior (Alley, 1983; Zebrowitz, 1997; Glocker et al., 2008). The underlying innate releasing mechanism also responds to childlike characteristics in young animals from other species, which is certainly one of the reasons why fluffy kittens and clumsy puppies make first rate kitsch subjects. In kitsch, infantile features are typically exaggerated (e.g., very large head with big round eyes) to make its bearers even more salient and adorable (peak shift principle; Ramachandran and Hirstein, 1999).^6^

Logically, a subject or theme has to be “instantly and effortlessly identifiable” (Kulka, 1996, p. 33) in order to elicit an automatic emotional response that is not deeply reflected. For the sake of immediate recognition, kitsch producers are well-advised to adhere to faithful imitation and to respect representational conventions (Figure 1A). A Cubist rendering, for example, will not work for kitsch as it impairs identifiability of the depicted subject matter (Muth et al., 2013). For example, Picasso’s rather unusual interpretation of *The Kiss* from 1928 fails to trigger an immediate heartwarming response despite its emotionally charged content (Figure 1B). This work from the artist’s Surrealist period shows that identifiability of the depicted subject matter was of little concern to him. On the contrary, Picasso deliberately complicated object recognition by transforming the romantic scene into what looks like a pile of bulky, perforated slabs of stone, while the maker of *First Kiss* (Figure 1A) chose a realistic display to ensure immediate identifiability.

**FIGURE 2:**
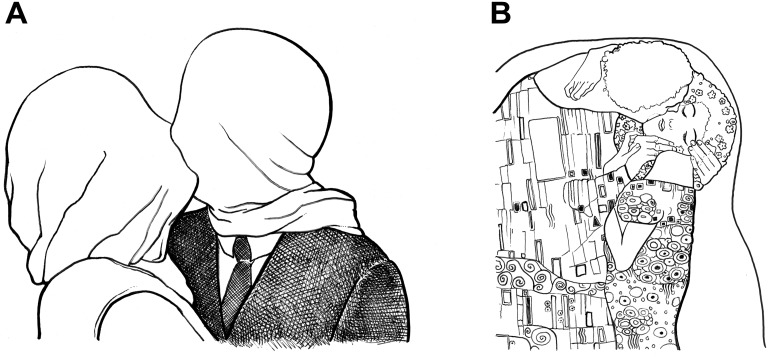
**(A)** *The Lovers* by Magritte (1928) and **(B)** *The Kiss/Lovers* by Klimt (1908). Abstracted drawings by the first author.

However, another Surrealist painting from 1928 titled *The Lovers* (Figure 2A) by René Magritte suggests that at least one more precondition is needed to reliably distinguish between kitsch and art. Although it deals with a positive emotional subject which is perfectly identifiable due to a realistic display, Magritte’s painting does not strike us as particularly kitschy. From the moment we lay eyes on it, we ask ourselves with puzzlement: Why are the lovers’ faces veiled? With this conspicuous detail the artist deliberately provokes conscious reasoning at the expense of an unreflective emotional response. Yet, the resulting train of thought may yield new interpretations (e.g., “By hiding the couple’s faces, the artist reveals the spectator’s voyeurism”). What do Picasso’s *Kiss* and Magritte’s *Lovers* have in common that is essentially absent in kitsch? From a modernist standpoint, art is valued for its ability to question our view upon the world (Armstrong and Detweiler-Bedell, 2008; Muth et al., 2015; Muth and Carbon, 2016). Indeed, the two artworks stand out from kitsch in that they challenge the habitual way of dealing with the depicted subject matter. Instead of following well-tried representational conventions, both Picasso and Magritte are testing the limits of artistic expression. Conversely, Kulka (1996) claimed that kitsch classification implies a manner of representation, which “does not substantially enrich our associations relating to the depicted subject matter” (p. 37). Alike Greenberg (1939) and many others, he arrived at the conclusion that kitsch meets a reassuring function, which is directly opposed to the intentions of the avant-garde: While art questions our basic sentiments and beliefs, kitsch comes to support and to protect them (Kulka, 1996).

Despite its conservative function, kitsch proves remarkably versatile. Greenberg (1939) described the paradoxical nature of kitsch—both static and dynamic—by stating that “[k]itsch changes according to style, but remains always the same” (p. 40). Novelty is an ephemeral property and artistic innovations are subject to adaptation on a cultural level. As society’s frame of reference shifts over time, even highly controversial artworks may gradually become clichés of high art and finally lend themselves to commercial exploitation. Today, museum shops sell coffee mugs, T-shirts, and pillow cases imprinted with reproductions of *The Kiss*/*Lovers* by Gustav Klimt (Figure 2B). Albeit the artist once spearheaded the Vienna Secession movement, innovativeness of his work has diminished and it has become particularly prone to kitsch classification: Apart from its abstract ornamental motifs, the highly emotional subject of this work is perfectly identifiable. It certainly adds to the plausibility of Kulka’s (1996) definition that commercial exploitation mainly affects figurative art, but not Cubist or non-representational artworks that lack an identifiable subject altogether. A corresponding habituation effect should also be discernible on an individual level. Since Kulka’s kitsch criteria are dynamically related to a person’s previous experience with a particular subject and its representational conventions, his definition can account for the variability that lies in the eye of the beholder: For example, some jolly little plastic figure with a beard and a pointed hat is certain to enrich someone’s associations who is unfamiliar with the concept of a garden gnome (e.g., children or foreigners). Yet, as novelty of the standard garden gnome decreases, it will gradually become prone to kitsch classification.

To summarize: By drawing on Kulka’s three conditions we can account for both the static and the volatile aspects of kitsch. As the “rear-guard” (Greenberg, 1939, p. 39) of cultural change kitsch readily appropriates new patterns after they have proved culturally successful. Nevertheless, it always stays the same with regard to its conservative function. Thus, Kulka’s (1996) definition allows us to take an interactionist perspective on kitsch (and art) as it groups “different objects […] together not because of their inherent similarities but because they fulfill a certain social function in a given society” (p. 6). But still a profound ambivalence toward kitsch remains unexplained: On the one hand “kitsch” is a term of abuse, on the other hand, it proves commercially successful.

## Why Is Kitsch Aesthetically Pleasing?

In *Changing Art, Changing Man* Mandel (1967) compared art galleries and museums to fitness studios of the mind where people enjoy exercising their spiritual abilities on works of art. By way of analogy, he suggested that Modern art is valued mainly for its capacity to transform the way we see the world. There is considerable empirical evidence that curiosity and exploration are in fact what motivates museum visitors to engage with art: Attending a show of contemporary art, people are “[e]xpecting the unexpected” (Muth et al., 2017a) and intrigued especially by works of art that promise new insights (Muth et al., 2015). When Berlyne (1971) formulated the basic propositions of *new experimental aesthetics* he had a similar notion of art in mind, according to which, *collative variables*^7^ such as novelty, surprise, complexity, indeterminacy, and ambiguity form not only the “essential ingredients of art [but] of whatever else is aesthetically pleasing” (p. viii). Yet, this assumption cannot account for the popular success of kitsch. What is it then that makes kitsch aesthetically pleasing?

In popular aesthetics form is subordinated to content (Bourdieu, 1979/1984). First and foremost, works of popular taste need a theme or a subject matter that “easily triggers a personal reflection or affiliation from spectators” (Hanquinet et al., 2014, p. 113). For the sake of immediate accessibility, its producers prefer well-tried manners of representation (e.g., conventional realism and the classical ideals of beauty and harmony) over daring stylistic innovations (e.g., Cubism; see Muth et al., 2013). As a result, everybody can easily relate to such works based on his or her common life experience. Obviously, kitsch makes a perfect example of popular taste in that it combines meaningful content with immediacy of a positive affective response. In Figure 1A, for instance, formal aspects are clearly subordinated to content as stylistic devices are either used to facilitate identifiability (i.e., faithful imitation) or to amplify the emotional impact (e.g., baby scheme) of a proper amalgam of emotionally rich content (e.g., first love).

The popular principle of ‘content over form’ led Kulka (1996) to believe that the mass appeal of kitsch must be determined solely by content-related associations: “People are attracted to kitsch because they like its subject matter” (p. 28). Are content-independent properties of kitsch really without any bearing on its hedonic value? After all, there is strong empirical evidence that any aspect of a stimulus array that helps the perceiver to process it more efficiently will also increase aesthetic liking. For example, it has been shown that people tend to prefer familiar over unfamiliar (Zajonc, 1968), clear-cut over indeterminate (Reber et al., 1998), and prototypical over unconventional stimuli (Rhodes and Tremewan, 1996; Halberstadt, 2006; Khalil and McBeath, 2006). From this body of research the authors of the *Hedonic Fluency Model* (HFM; Reber et al., 2004) concluded that liking is a monotonically increasing function of processing speed: “The more fluently the perceiver can process an object, the more positive is his or her aesthetic response” (p. 366).^8^ Fluent processing is hereby conceived as an inherently pleasant experience (Reber et al., 2004) spilling over onto the stimulus itself (Winkielman and Cacioppo, 2001). Besides, Reber et al. (2004) distinguished *perceptual fluency*, that refers to “the ease of identifying the physical identity of the stimulus” (p. 367), from *conceptual fluency*, which is defined as the “ease of mental operations concerned with stimulus meaning and its relation to semantic knowledge structures” (p. 367). In terms of processing ease, we expect kitsch to excel on a perceptual and a conceptual level: According to Kulka’s (1996) definition, kitsch classification directly implies effortless identifiability and standard associations with regard to the depicted subject matter.

Altogether, we hypothesize that the aesthetic appeal of kitsch consists of (A) emotionally rich content with a positive valence in combination with the inherently rewarding experience of (B) perceptual (effortless identifiability), and (C) conceptual fluency (standard associations). Jointly, these three components offer a reasonable explanation for its great popularity: Kitsch is liked simply because it provides instant and effortless emotional gratification (see Benjamin, 1982/2002; Cǎlinescu, 1987; Kulka, 1996; Menninghaus, 2009; Ortlieb and Carbon, 2018). When we talk about kitsch and art it seems that we are dealing with two different kinds of aesthetic appreciation that can be reliably separated in terms of processing dynamics (Graf and Landwehr, 2015) and the role of positive content-related associations (Ortlieb and Carbon, 2018): a fluent one, consisting of a spontaneous, inherently pleasurable affective response and general accessibility (kitsch); and a disfluent one, that may yield new insights but requires previous knowledge and cognitive elaboration (art; Belke et al., 2015).

## Why Is Kitsch Considered Worthless?

If kitsch is perfectly agreeable, why is it a derogatory term in the first place? With his *Social Critique of the Judgement of Taste* sociologist Pierre Bourdieu raised awareness for “a fundamental refusal of the *facile*” (Bourdieu, 1979/1984, p. 486) in Western aesthetics. This general aversion is directed against anything that appears “easy in the sense of simple, and therefore shallow, and ‘cheap,’ because it is easily decoded and culturally ‘undemanding”’ (p. 486). Furthermore, he observed that whatever “offers pleasures that are too immediately accessible [is contrasted with] the deferred pleasures of legitimate art” (p. 486). Although Bourdieu did not mention kitsch explicitly, it makes a truly paradigmatic example of his claims: On the one hand, it is liked inter alia because it is easy on the mind; on the other hand, it is used derogatorily by art-educated people in contrast to high art.

Highbrow aesthetics is directly opposed to popular taste in that it “privileges form over matter and the principle of distance and detachment in the appreciation of art” (Hanquinet et al., 2014, p. 113). An artwork is seen as autonomous in that it reflects the artist’s idiosyncratic message without making any concessions to common understanding. These characteristics of highbrow aesthetics are discernible in Picasso’s *Kiss* (Figure 1B): Apparently, the artist is more interested in formal experimentation than in the emotional content of his work. At the expense of identifiability and immediacy of effect, he seeks for hitherto unprecedented means to express himself. Bourdieu et al. (1969/1991) argued that highbrow aesthetics “cannot be appreciated immediately without any cultural resources” (Hanquinet et al., 2014, p. 113). In order to apprehend and value high art, the perceiver has to acquire *culture capital* (e.g., art expertise), “a resource which is unevenly socially distributed” (p. 113). Therefore, Bourdieu’s “distinction between popular aesthetic and highbrow aesthetic is […] one between the initiated and outsiders, between the few that master the aesthetic codes and are able to decipher them and the many others who belong to the profane world” (p. 113). Since aesthetic ideas have been shaped in a social process, they bear the mark of relationships of power and domination (Hanquinet et al., 2014). From this premise, Bourdieu (1979/1984) concluded that a “refusal of the *facile*” (S. 486) is maintained, because popular taste does not allow for distinction in terms of culture capital. In other words, social inequality could neither be secured nor ‘legitimized’ based on popular aesthetics.

Consequently, the doctrine of Socialist Realism of the former Soviet Union obliged artists to refrain from abstraction and formal experimentation (formalism) in favor of a realistic representation of themes from everyday life. In a classless society, art should be intelligible for everyone. According to the composer György Ligeti, this culture policy inevitably led to “cheap mass-produced art” (Ligeti, 2010). Alike other art doctrines, Socialist Realism illustrates how art becomes kitsch, whenever it is stripped of its dishabituation function and used as a vehicle for political propaganda (Kundera, 1984/1999). Culture capital also offers a plausible explanation why some formerly derogatory concepts have become highly respectable terms (e.g., Impressionism), while kitsch continues to be negatively connoted. An Impressionist painting is unlikely to please the naïve spectator at first sight: Its subject is only vaguely identifiable as brushstrokes have been hastily jotted onto the canvas. To the naïve perceiver it appears unfinished or otherwise carelessly executed. Some background knowledge (i.e., culture capital) about the intentions, practices, and merits of Impressionist painting is required to fully appreciate these works (Kulka, 1996). By contrast, in the case of kitsch aesthetic pleasure is without any presuppositions in terms of art expertise—most people can relate to it at first sight. Research literature from empirical aesthetics supports Bourdieu’s assumption by showing that a transfer of art-related knowledge increases people’s appreciation of novelty and complexity in general (McWhinnie, 1968; Smith and Melara, 1990; Palmer and Griscom, 2013) and of abstract paintings in particular (Stojilović and Marković, 2014). At least two of these studies even suggest that an increase in aesthetic expertise leads to a veritable distaste for conventional harmony (Smith and Melara, 1990; Palmer and Griscom, 2013).

If we think of kitsch and avant-garde art as the poles of a continuous dimension connecting perfectly conventional, benign, and determinate aesthetic objects (kitsch) and highly original, ambivalent, and indeterminate ones (avant-garde art), one’s current level of art expertise defines the anchor point of one’s personal aesthetic frame of (p)reference. With an increasing level of art expertise this set point moves toward the avant-garde pole. Thus, the range of people’s aesthetic comfort zone will vary greatly according to their aesthetic standards. As a result, Monet’s famous water lilies may provoke extremely different reactions: They may be too concrete and mainstream for some, while others dismiss them as too abstract and avant-gardist. By and large, the concept of culture capital can account for such interindividual differences in aesthetic judgment; yet it does not explain why the same person is not always ‘in the mood’ for Monet. On the whole, Bourdieu’s view on taste is a rather static one. However, the value of a sociological perspective should not disguise the fact that our relationship to kitsch and art is more complex and flexible. For instance, it cannot explain why, from time to time, people are attracted to kitsch rather than repulsed by it (Kulka, 1996; Kundera, 1984/1999; Dettmar and Küpper, 2007; Pazaurek, 1912/2012). In the following we devise a theoretical framework that accounts for the dynamic aspects of aesthetic liking by relating them to the ever-conflicting demands of human attachment behavior.

## Linking Kitsch and Art to the Dynamics of Social Motivation

“I call beauty a social quality”

(Burke, 1757/1990, p. 39)

Philosopher Walter Benjamin once claimed that “art begins at a distance of two meters from the body [while] in kitsch, the world of things advances on the human being” (Benjamin, 1927/2008, p. 238). As we will see in the following this is by no means a metaphorical expression, but a very accurate observation which can be taken almost literally: By relating changes in aesthetic liking to the dynamics of social distance regulation, we will devise a functional model of kitsch and art that rests on two propositions: Firstly, kitsch and avant-garde art ideally represent two types of aesthetic experience, which can be reliably discriminated in terms of processing characteristics and positive emotional content; secondly, preference for the one or the other is modulated by needs for intimacy and autonomy. Similar claims have been made by philosopher Edmund Burke who already speculated about a close interrelation between two distinct aesthetic ideas—the beautiful and the sublime—and their origins in social motivation (Burke, 1757/1990). According to Burke, it is a drive for affiliation that attracts us to the beautiful, while a drive for self-preservation fascinates us with the sublime: Anything beautiful evokes tender feelings of affection along with the desire to draw near to it (e.g., fluffy kittens). In contrast, the underlying emotions of the sublime are fear and awe (Eskine et al., 2012). Hence, the sublime is only aesthetically pleasing as long as it is observed from a safe distance (e.g., tigers at the zoo). By proposing two antagonistic drives for affiliation and self-preservation Burke’s theory touches upon the basic dilemma of social distance regulation that is vividly illustrated by Schopenhauer’s (1851/1989) well-known ‘porcupine parable’: On a cold day a group of porcupines huddles together in search of warmth; yet the closer they move together, the more they hurt each other with their spines. As a result, they veer away from each other until the need for warmth prevails and they search each other’s company again. Now this “primordial conflict of intimacy and autonomy” (Bischof, 1975, p. 1) is not only inherent in porcupines, but also in humans and other social animals. Moreover, the basic dilemma of social motivation has always been an inexhaustible source of inspiration for artistic production: Apart from leaving its universal mark on fairy tales and world mythology (Campbell, 1949; Bischof, 2004), it is the stuff of poems, novels, and theater plays, as well as pop music, films, and daily soaps. We therefore make a case that dynamics of social motivation are key to understanding the changes in aesthetic appreciation on a cultural, an individual, and a situational level. For the sake of clarity, we devise our model in a stepwise approach starting with the four basic components of the *Zurich Model of Social Motivation* (Bischof, 1975, 1993, 2001). In a second instance, we introduce the concepts of self-efficacy (Bandura, 1977) and regulatory focus (Higgins, 1998) to the model. In doing this we shall point out how each of these variables can be related to concepts and findings from empirical aesthetics. Finally, we hypothesize how needs for intimacy and autonomy modulate aesthetic preferences based on two scenarios.

### Zurich Model of Social Motivation

The *Zurich Model of Social Motivation* by Bischof (1975, 1993, 2001) is a comprehensive systems theory of social distance regulation in humans and other social animals, including porcupines. Its key proposition reads that human attachment behavior is hinged upon two antagonistic motives for intimacy and autonomy. According to Bischof, it takes at least three different motivational systems to deal with the complex requirements of social distance regulation comprehensively: an arousal system, a security system, and an autonomy system. In addition, he postulated an auxiliary system—the coping system—that only ‘kicks in’ if one of the other subsystems is blocked by an obstacle (Schneider, 2001). Since requirements of social motivation vary across the lifespan—attachment and protection are vital for the newborn, while autonomy and exploration are of utmost concern to the adolescent—the model also has developmental implications. In the following, the role of each subsystem is described and the close interrelations with concepts and findings from empirical aesthetics are pointed out. We start with the two subsystems that figure most prominently in early infancy: the arousal and the security system.

#### Arousal System

**FIGURE 3:**
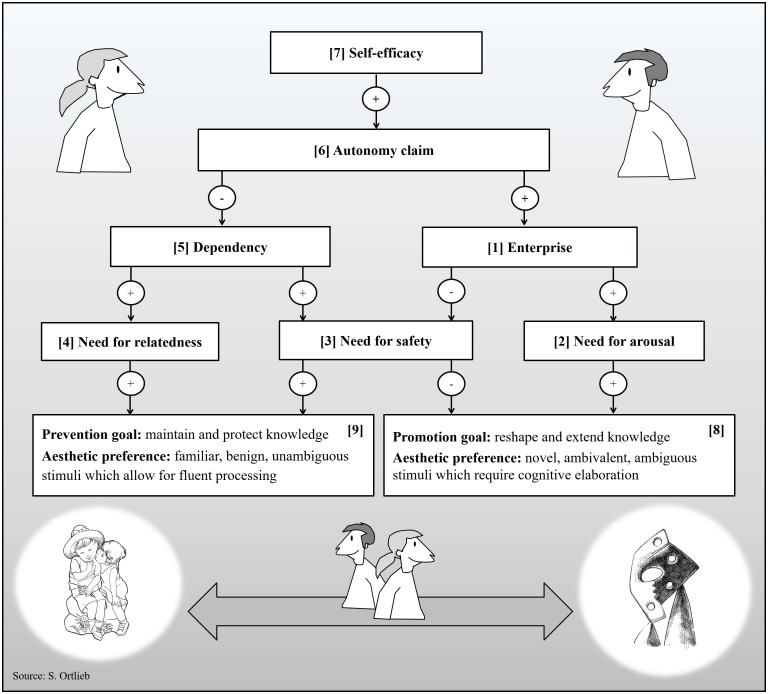
Overview of variables and interrelations. Arrows with a “+” indicate an excitatory relationship (e.g., if variable **[1]** is high, variable **[2]** is elevated), while arrows with “–” signify an inhibitory relationship (e.g., if variable **[1]** is high, variable **[3]** is diminished).

The arousal system monitors and regulates an individual’s current level of activation. Arousal refers to an unspecific activation pattern of the sympathetic nervous system that accompanies “interest, fascination, curiosity, as well as feelings of alarm or fear” (Schneider, 2001, p. 10122). It is based on the assumption that any unfamiliar event will provoke a (startle) response: When we walk the streets alone at night and suddenly notice the silhouette of a tall stranger, our heartbeat quickens involuntarily. Apart from enhancing our responsiveness to unexpected and potentially unpleasant encounters, the arousal system is also responsible for maintaining a level of basic activation. Whether the current state of activation is agreeable or not, depends on an internal reference variable called “enterprise” (see [1] in Figure 3). The current arousal level is continuously monitored in relation to this set point: If activation should fall short of enterprise, an appetence for arousal arises (see [2] in Figure 3): We are likely to show exploratory behavior in search for something excitingly new (curiosity). Conversely, whenever arousal exceeds enterprise, we experience sensory overload resulting in a temporary aversion to further collative stimulation (distress). For the arousal system, anything unfamiliar is associated with an increase in arousal. Thus, its activity is highest when a high-ranking adult stranger is approaching. The easiest way to maintain an agreeable level of arousal is to adjust one’s physical distance to this conspecific: In the case of an unexpected nightly encounter, we could change the side of the street or, should a state of curiosity prevail, walk toward the person and say hello.

The arousal system reflects a basic “need for exploration” (Lorenz, 1943) that has been related to a wide variety of universal cultural phenomena such as game, competition, and humor. Moreover, it has been argued that curiosity and exploratory behavior form the motivational basis for creating and responding to art (Dissanayake, 1990). In *Aesthetics and Psychobiology*, Berlyne (1971) devised a psychobiological model of aesthetics that draws on the same homeostatic concept of arousal management as the Zurich Model: By assuming that the hedonic quality of an artwork lies in its arousal potential, Berlyne postulated a positive link between the aesthetic appeal of a stimulus array and its degree of novelty, complexity, uncertainty, and ambiguity. Since random patterns are, however, not considered aesthetically pleasing, liking is not seen as a monotonically increasing function of collative stimulus properties. According to Berlyne, the relationship between the hedonic (i.e., aesthetic) value and the arousal potential of a stimulus array is expected to follow an inverted u-shaped curve. The maximum of this curve should vary, depending on a viewer’s previous experience: For example, the more art expertise one has, the more unlikely it is that one encounters something surprisingly new and discrepant. By stating that aesthetic appeal is a function of arousal potential, Berlyne’s notion of aesthetics is perfectly in line with the core values of avant-garde art: novelty and change. This emphasis on novelty and conflict, however, disregards aesthetic pleasure associated with fluent processing. Especially, the popular success of kitsch and a widespread indifference toward Modern art cast doubt on Berlyne’s claims. A second motivational system is needed to account for the “warm glow of familiarity” and conventional harmony.

#### Security System

Probably the most important variable for attachment behavior is security (Bowlby, 1969). It can be defined as a feeling of warmth and protection that is instilled by the presence of familiar and trustworthy individuals (Schneider, 2001). Security hereby combines notions of intimacy and safety. Therefore, the security system regulates a need for safety (see [3] in Figure 3) as well as a need for relatedness (see [4] in Figure 3). Otherwise, it works analogously to the arousal system: In the case of the security system the reference variable is called “dependency” (see [5] in Figure 3). Yet in contrast to the arousal system, activity of the security system peaks whenever a familiar and relevant conspecific is close (e.g., during infancy this is typically one’s primary caregiver). Our notion of security is continuously monitored with regard to this set point: “As long as dependency exceeds security, needs for safety and relatedness are maintained. This induces the subject to show attachment behavior, that is, to reduce the distance to a person who is able to provide security. The opposite situation, frequently encountered in puberty, results in an aversion against security and consequently in an avoidance of familiar persons (surfeit behavior)” (Schneider, 2001, p. 10122).

The security system of the Zurich Model accounts for a large body of research on attachment behavior inspired by the seminal works of John Bowlby (1969). Also with reference to Bowlby’s attachment theory, Dissanayake (2015) has made a case that “the close early interactions between infants and their caretakers are the prototypes for what will become our later experiences of love, allegiance, art, and other forms of self-transcendence” (p. 7). In *Art and Intimacy*, she argued that human sensibility for aesthetic experience arises from our extraordinary responsiveness to social cues that in turn results from the vital significance of attachment in early infancy. From an evolutionary perspective it must be inherently pleasurable to care for a baby and to establish and maintain close social bonds between babies and their primary care-givers (e.g., physiologically triggered by oxytocin release or perceptually by the baby scheme). Conversely, it is of vital interest to the baby to memorize the individual characteristics (e.g., sound of the voice, smell, facial features) of the people it frequently engages with (Bischof-Köhler, 2006). Since there is no possibility for a newborn baby to identify its biological parents among other adults, frequent exposure (i.e., familiarity) seems the best estimate. The more often an individual is around, the more likely it is a caring, trustworthy kin. It is therefore quite reasonable to assume that a general preference for familiar stimuli (Zajonc, 1968) originates in a hard-wired heuristic equating familiarity with safety (Smith, 2000; Zajonc, 2001) and relatedness. Due to its vital importance, especially during infancy, this mechanism is not limited to conspecifics; it also spills over onto places (e.g., one’s birthplace) or inanimate objects (e.g., heirloom, keepsakes, talismans, and souvenirs).^9^ In several respects, kitsch actually seems to mimic the characteristics of familiar and trustworthy conspecifics, typically represented by primary caregivers: It is familiar, unambiguous, and elicits positive emotions (e.g., mother with child, baby scheme).

In a now classical textbook of psychology, Titchener (1910) wrote that recognition of familiar stimuli is associated with “a glow of warmth, a sense of ownership, a feeling of intimacy, a sense of being at home,” (p. 408) as well as feelings of ease and comfort. This definition is well in line with the hypothesis that familiarity is an ecologically valid cue for safety (Smith, 2000; Zajonc, 2001) and that “positive affect is [therefore] integral to the implicit feeling of familiarity” (Garcia-Marques and Mackie, 2001, p. 241). Research on the so-called “mere-exposure effect” (Zajonc, 1968) shows that liking is positively correlated with exposure rates; a finding which is not limited to the visual domain but expands to other modalities as well (haptics; Jakesch and Carbon, 2012). In a cross-cultural study on kitsch, a positive interrelation between self-transcendence and liking of decorative everyday objects was found (Ortlieb et al., 2017). Another rating study using the same stimulus material in combination with the *Motive Profile Following the Zurich Model* (MPZM; Schönbrodt et al., 2009) showed that decorative day-to-day objects were perceived as less kitschy and more likable by participants who valued security over arousal (Vlasova et al., 2018). As already mentioned by Bischof (2001), the security system also forms the psychobiological basis for xenophobia. According to the Zurich Model, a need for security is inherently related to an aversion of arousal. Since unfamiliar conspecifics constitute a source of arousal, a need for security will inevitably increase likeliness for xenophobic reactions (e.g., fear of strangers in infancy). Interestingly, this ambivalence is also found in kitsch that readily lends itself to right-wing propaganda (Friedländer, 1985/2007). Schmidt (1994), for example, observed that “something in kitsch refers to homeland and familiarity, a need which cannot be rejected, but which has to be mistrusted” (p. 143).

#### Autonomy System

Autonomy refers to freedom from external control (independence) and the capacity to act in accordance with one’s own rules and principles (self-determination). Since dependency upon adult caretakers is very high throughout childhood it is not until early adolescence that the third motivational system—the autonomy system—comes into play. To what extent we aspire autonomy (or avoid it) is determined by the autonomy system and its reference variable “autonomy claim,” (see [6] in Figure 3) which “is closely related to the power and the achievement motives, as well as the need for prestige and an aspiration for self-confidence” (Schneider, 2001, p. 10123). The autonomy system receives its input from a different source than the other two systems. A person’s autonomy claim increases with his or her notion of achievement: When we succeed in solving problems and obtain other people’s admiration or acknowledgments our notion of self-confidence increases (Schneider, 2001). The Zurich Model posits that whenever “individuals feel an appetence for autonomy, they are assumed to behave assertively by becoming threatening, demanding, or even aggressive. In the opposite case, if individuals have too much autonomy and feel aversive, they will show submissive behaviors such as behaving humbly or servilely” (p. 10123). Among the three subsystems of the Zurich Model, the autonomy system takes an exceptional position. Its reference variable (autonomy claim) modulates the set points of the other two subsystems (dependency and enterprise) in that it inhibits dependency and enhances enterprise: If self-confidence is diminished, autonomy claim will also be reduced. As a consequence, needs for safety and relatedness stand a good chance to prevail over a need for arousal. In the opposite case, whenever autonomy claim is increased due to a high level of self-efficacy, a need for arousal is likely to arise, while needs for safety and relatedness are tuned down. Interestingly, Bischof (2001) assumes that the activity of the autonomy system is inhibited during infancy and that its reference variable (autonomy claim) peaks during puberty. Thus, if aesthetic appreciation is related to an urge for autonomy, we would expect a preference for arousing stimuli to be particularly pronounced among adolescents.

From an art historian’s point of view, autonomy is a key concept to modern aesthetics. On the whole, the historical avant-garde has been described as an emancipatory movement aiming for a “breakup of traditional aesthetic authority” (Cǎlinescu, 1987, p. 4). Inspired by Kant’s (1790/1951) distinction between free beauty (*freie Schönheit*) and dependent beauty (*anhängende Schönheit*), modern artists struggle for an individual diction and against the aesthetic conventions of the past.^10^ In the second half of the 19th century this autonomy claim became manifest in the unprecedented idea of *art for art’s sake* as well as in alternative art institutions such as the Societé des Artiste Indépendants in Paris or the Viennese Secession. This detachment from normative constraints also redefines the role of the perceiver, who is now expected to maintain a critical distance instead of readily indulging in beauty and harmony. As has been pointed out before, the derogatory label of kitsch plays a significant role in intergenerational conflict: Artists and spectators who expressed “nostalgic feelings about the lost ancient ideal of beauty” (Cǎlinescu, 1987, p. 4) risked to be scorned as “Kitschiers” (Avenarius, 1920, p. 222).

#### Self-Efficacy

In the previous section it has been stated that the set point of the autonomy system (autonomy claim) is positively linked to one’s personal sense of achievement and competence. For conceptual clarity, we identify this variable with self-efficacy (see [7] in Figure 3). Following Bandura’s (1977) classical definition, self-efficacy refers to the extent of a person’s confidence in his or her own ability to complete tasks, solve problems, and achieve goals. Usually, notions of self-efficacy vary across different situations depending on a person’s skills and his or her previous experience with certain tasks. Perceived self-efficacy is generally higher in familiar, non-threatening situations than in unfamiliar and potentially threatening ones. According to our model, different notions of self-efficacy have a direct bearing on a person’s autonomy claim: Whenever we enjoy a high sense of competence and achievement, we are likely to feel more self-determined and thus less dependent on others.

How does self-efficacy affect aesthetic preferences? If self-efficacy is positively linked to the reference variable of the autonomy system (autonomy claim), which, in turn, defines the set points of the security (dependency) and the arousal system (enterprise), then it should also modulate aesthetic preferences for conventional, clear-cut, and benign stimuli, or original, indeterminate, and ambivalent ones, respectively. Findings by Muth et al. (2017a) suggest that “[t]he experience of ambivalent images is strongly linked to mood and self-efficacy” (p. 307): Muth and her colleagues observed that participants rated artistic photographs with highly ambivalent content more positively, if they had received encouraging feedback on a previous puzzle task.

In one of the above sections (Why Is Kitsch Considered Worthless?) we have found that art expertise plays an eminent role when it comes to judgments of taste. Art expertise can be interpreted as a task-related component of self-efficacy in that it gives us the feeling that we can account for our aesthetic judgments based on valid criteria. As mentioned before, there is substantial empirical evidence that art expertise tends to increase people’s preference for stimuli which stand out from their previous experience. Based on a review of studies on perceptual choices in artists with non-artists, McWhinnie (1968) concluded that “[i]ndividuals with training in art seem to prefer the more complex figures; whereas, those without training prefer the simple figures” (p. 373). Concordantly, Stojilović and Marković (2014) observed that art lectures increase appreciation of abstract paintings and with regard to music, Smith and Melara (1990) found that music graduate students preferred atypical harmonic progressions, while novices favored music with conventional harmonies. Palmer and Griscom (2013) also examined preferences for conventional harmony using four different sets of stimuli (color, shape, spatial location, and music). They reported that individual preferences for harmony were highly correlated across these domains. Yet this initial preference for harmonious stimuli “decreased consistently with training in the relevant aesthetic domain” (p. 453). As a task-related component of self-efficacy, art expertise clearly seems to elevate people’s aesthetic standards with regard to complexity and originality.

Theoretically, one’s current level of art expertise defines the anchor point on a continuous dimension connecting the polar opposites of easy-to-process aesthetic objects (kitsch) and difficult-to-process ones (avant-garde art). The more art expertise we command, the closer our anchor point will be to the avant-garde pole and vice versa. Wherever our frame of (p)reference may be located on this dimension, we expect to observe the following dynamics relative to this individual anchor point: Prevalent needs for safety and relatedness will shift the aesthetic comfort zone away from the initial set point into the direction of the kitsch pole, while needs for arousal and autonomy will push it toward the avant-garde pole. In the following we make a case that these tendencies should be particularly pronounced, whenever the default mechanism of social distance regulation is unavailable to us and we have to deal with anxiety (or boredom) symbolically.

#### Coping System

“Kitsch is the quickest means of reconciling oneself to circumstances”

(Schmidt, 1994, p. 141)

How do we accommodate needs for security and arousal when physical distance regulation is ruled out? Often we cannot simply walk away from a source of distress (or tedium) and when we feel miserable there is no guarantee that friends and relatives are available to comfort us. Should physical distance regulation be blocked by an obstacle, the auxiliary system of the Zurich Model comes into play. The so-called coping system serves as a kind of toolbox for emergency situations. It contains three sets of reactions: aggression, supplication, and invention.

Aggression and supplication are probably the most primordial responses to critical situations. Supplication means that one turns to another person for help. This is the first and one of the most effective coping strategies (apart from invention): A baby, for example, has no choice but to send out supplication signals to its care-givers. Thanks to the aforementioned baby scheme and the corresponding innate releasing mechanism, supplication signals are in fact inscribed in the baby’s bodily appearance promoting caretaking behavior and inhibiting aggression (Zebrowitz, 1997). In early infancy, it is mainly the primary care-givers who provide a safe and optimally stimulating environment: Either by preventing overstimulation or by engaging in lively face-to-face interaction (Dissanayake, 2015). As soon as an infant is capable of crawling, however, it starts to self-regulate needs for arousal and security via locomotion and eye-contact with its caregivers. Finally, with language acquisition toddlers learn that symbols and signs may serve as safety signals. Symbols carrying cultural or idiosyncratic meaning offer new possibilities to deal with trying situations: For example, it is through rituals, talismans, keepsakes, and lucky charms that people bolster their notions of security and achievement. Many objects we find on office desks bespeak these two needs: Family photos convey feelings of affection as they emulate the people dearest to us, while sport trophies and diplomas work as a source of pride and self-confidence by reminding us of past achievements (Csikszentmihalyi, 1996; Norman, 2004; Csikszentmihalyi and Rochberg-Halton, 1981). According to Fischer et al. (2018), there is preliminary indication that people with different anxiety-related coping styles respond differently to everyday things: Participants with little confidence in their own abilities who claimed to be rather intolerant of uncertainty and highly vigilant about threatening information (*sensitizers*) rated decorative objects more likable and less kitschy than participants who overestimated their abilities and habitually avoid or deny threatening cues (*repressors*). This complementary preference pattern in sensitizers and repressors directs our attention to the final component of our model: regulatory focus.

### Regulatory Focus

A systems theoretical approach to dynamics of aesthetic liking implicates that aesthetic evaluation is somehow goal directed and therefore regulated by feedback-controlled processes. *Regulatory Focus Theory* by Higgins (1998) proposes a fundamental distinction between two motivational orientations: One directing us toward preventing threats (*prevention focus*) and another one that promotes opportunities for growth and achievement (*promotion focus*). A prevention focus clearly reflects a need for safety in that it increases our sensitivity to possible threats in our environment. Besides, it motivates us to protect and maintain our present knowledge structures. By contrast, a promotion focus is rooted in a need for learning and achievement that closely resembles an appetence for arousal (curiosity): It increases our sensitivity to opportunities rather than to potential risks. Thus, a promotion focus entails the urge to extend or at least modify one’s present knowledge.

How does regulatory focus relate to aesthetic liking? Armstrong and Detweiler-Bedell (2008) have distinguished between “mild aesthetic pleasure associated with simple or familiar objects [and a] more intense pleasure associated with complex or novel objects” (p. 305). Inspired by Kant’s (1790/1951) opposition of dependent and free beauty they claimed that these two forms of aesthetic pleasure can be differentiated in terms of regulatory focus and corresponding epistemic goals: “Pretty, fluently processed stimuli implicate prevention goals that maintain and project knowledge. Beautiful, novel stimuli implicate promotion goals that reshape and expand knowledge” (Armstrong and Detweiler-Bedell, 2008, p. 305). Apparently, this distinction fits squarely to the concepts of kitsch and Modern art advocated in the present article: Kitsch, it seems, is designed to please prevention goals, while avant-garde art promises promotion goal attainment. There is also empirical evidence that regulatory focus modulates aesthetic liking of easy-to-process stimuli. In an experiment by Freitas et al. (2005) the motivational context in which people experienced fluent processing was manipulated. One group of participants was instructed to describe strategies for the attainment of good health, good grades, and financial success (promotion condition), while another group was asked to generate strategies for avoiding health problems as well as academic and financial failure (prevention condition). Subsequently, all participants evaluated images of affectively neutral, everyday objects that were presented either with matching (fluent stimuli) or mismatching (non-fluent stimuli) contour primes. As a result, only participants from the prevention condition showed a preference for easy-to-process stimuli. Together with findings from another concordant study these results amounted to the conclusion that “safety connotations of familiarity are valued in relation to one’s current motivational orientation” (p. 642). It seems that hedonic value of fluent processing is moderated by contextual factors as well as by initial stimulus valence (Albrecht and Carbon, 2014). Since we have claimed that immediate identifiability is one of the most important assets of kitsch, we expect that regulatory focus and appreciation of kitsch are dynamically interrelated. In the next section, we take a closer look at the system dynamics.

### Dynamics of Aesthetic Appreciation

Finally, the variables of our model are compiled and their dynamic interplay is described on the basis of two complementary scenarios. For each scenario additional empirical evidence is presented to elaborate on this dynamic approach to aesthetic liking.

#### Scenario I: Familiarity Breeds Contempt

**FIGURE 4:**
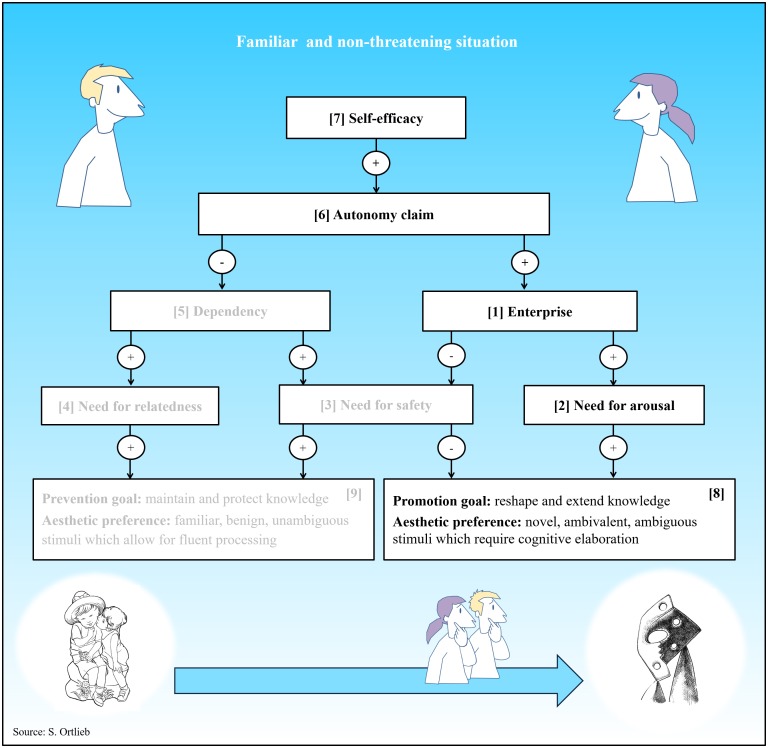
System dynamics in a familiar and non-threatening situation. Arrows with a “+” indicate an excitatory relationship (e.g., if variable **[1]** is high, variable **[2]** is elevated), while arrows with “–” signify an inhibitory relationship (e.g., if variable **[1]** is high, variable **[3]** is diminished).

Figure 4 illustrates the system dynamics for a familiar and non-threatening situation. Whenever the environment is safe and predictable, people feel more self-sufficient as they enjoy a higher level of self-efficacy. Under such conditions, the reference variables of the autonomy system (autonomy claim) and the arousal system (enterprise) are increased, while the reference variable of the security system (dependency) is tuned down. Given these parameters, an appetence for arousal easily overrules needs for safety and relatedness. Consequently, the model predicts a promotion focus, which means that a person is motivated to reshape and extend his or her previous knowledge (curiosity). With regard to aesthetic liking, this person’s frame of preference is also expected to move away from the initial anchor point into the direction of the avant-garde pole: He or she should show more interest in novel, complex, and ambiguous stimuli promising new insights (Armstrong and Detweiler-Bedell, 2008); i.e., qualities typically found in avant-garde art and cutting-edge design. In effect, there is empirical indication for a concordant interrelation between feelings of safety and the appreciation of innovative design (Carbon et al., 2013).

What happens if we fail to attain a promotion goal, because we encounter familiar easy-to-process stimuli instead? According to Armstrong and Detweiler-Bedell (2008), we are likely to experience boredom or even dejection since “failure to attain promotion goals leads to low-arousal, negative emotions because when a gain is unrealized, there is an absence of novelty—we are left with what we have, with what we know” (p. 317). When we seek for accomplishment and encounter nothing but perfectly conventional aesthetic objects with a strong emotional charge, we dispraise of them as kitsch. In a series of experiments De Vries et al. (2010) used self-reports and psychophysiological measures to explore whether cognitive and affective responses to familiar stimuli are modulated by mood. Participants were instructed to describe either a happy, or a sad autobiographical memory, before responding to a set of familiar (i.e., prototypical) and unfamiliar (i.e., non-prototypical) visual patterns. Participants in a sad mood showed a preference for familiar patterns. This was, however, not the case for participants in a happy mood: Although prototypical patterns were rated as more familiar, they were not valued more positively. From this study De Vries et al. (2010) concluded that“[i]f mood signals a safe environment, familiarity loses its glow” (p. 325). However, these findings also suggest that the opposite is the case when people feel troubled.

#### Scenario II: Home Sweet Home

**FIGURE 5:**
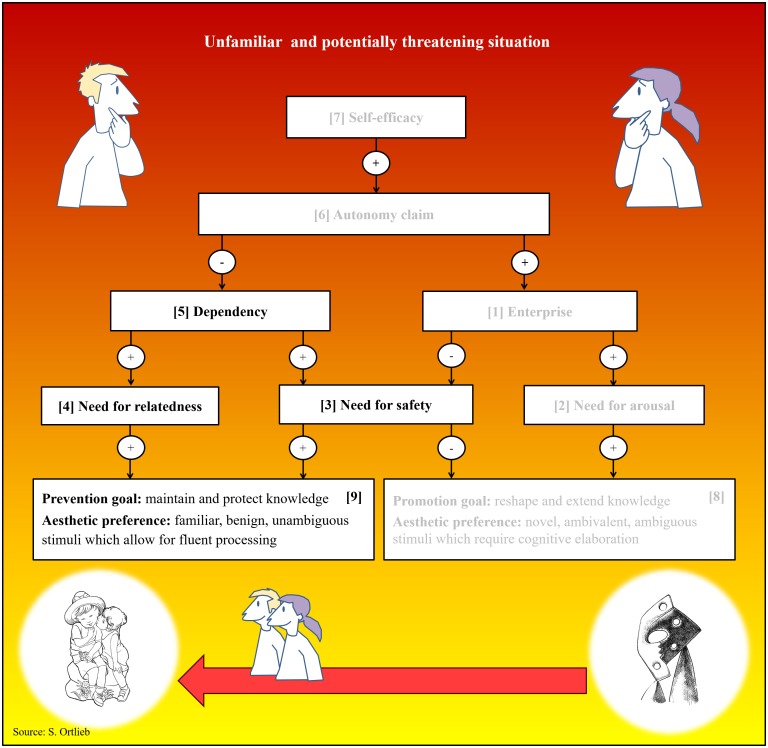
System dynamics in an unfamiliar and potentially threatening situation. Arrows with a “+” indicate an excitatory (e.g., if variable **[1]** is high, variable **[2]** is elevated), while arrows with “–” signify an inhibitory relationship (e.g., if variable **[1]** is high, variable **[3]** is diminished).

Imagine a group of pupils about to take their final exams. Some have brought stuffed animals or other lucky charms with them and placed them on their desk. The mere presence of these familiar and trusted objects seems to ease their inner tension in the face of a highly relevant and thus potentially threatening situation. Figure 5 shows the predicted system dynamics for such a trying situation: Under unfamiliar, uncertain, and potentially threatening conditions self-efficacy expectations are reduced. One’s autonomy claim is tuned down as one feels more vulnerable and dependent on others. With needs for safety and relatedness coming to the foreground, an aversion for arousal arises. Cognitively, this state is characterized by a prevention focus, implying a motivation to maintain and protect one’s previous knowledge (nostalgia). Accordingly, people’s aesthetic comfort zone will shift away from the initial set point toward the kitsch-pole; we thus expect people to become more susceptible to the familiar, clear-cut, and comforting properties of aesthetic objects they might otherwise dispraise as overly sentimental and consoling. Two experiments showed that decorative everyday objects were rated less kitschy, after participants had reflected on their own mortality (Raab et al., 2015; Ortlieb et al., 2016a).

What if we are confronted with strange, difficult-to-process aesthetic objects in pursuit of a prevention goal? Presumably, this causes a state of irritation and uneasiness due to an excessive increase in arousal. Feeling somewhat “lost in a chaos of sound and rhythms, colors and lines, without rhyme or reason” (Bourdieu, 1979/1984, p. 2), we expect the perceiver’s openness and affection for avant-garde art to be diminished. Accordingly, Landau et al. (2006) observed a more pronounced distaste for Modern artworks—representational and abstract paintings—in participants with a high need for cognitive structure after mortality concerns had been induced.

## General Discussion

“How does common taste change? Through individuals—powerful, influential, and without any sense of shame—who announce and tyrannically enforce [...] the judgment of their taste and disgust”

(Nietzsche, 1882/2001, p. 56)

The aim of the present paper is to show that dynamics in aesthetic liking are synchronized with a basic mechanism of social distance regulation that has evolved in social living animals to reconcile needs for safety and intimacy with needs for arousal and autonomy. We believe that it is this constant tension between attachment and detachment that creates emotional involvement and shapes the ‘ups and downs’ in interpersonal relationships as well as in aesthetic experiences. In modern Western aesthetics this conflict between tradition and innovation has produced the particularly clear-cut dichotomy of kitsch and avant-garde art that can be mapped onto two complementary streams of research in empirical aesthetics: Hedonic fluency (e.g., Reber et al., 2004) and cognitive mastery (e.g., Leder et al., 2004). Our model posits that preference for the one or the other is driven by needs for secure relatedness (nostalgia), respectively self-determined exploration (curiosity): Whenever we feel safe and self-sufficient, an appetence for excitingly new, complex, and ambiguous stimuli arises (art). By contrast, whenever we feel vulnerable and dependent, an aversion toward arousal emerges and we develop an appetence for safety and relatedness, which makes us susceptible to the warm glow of familiar, clear-cut, and benign stimuli (kitsch). According to our model, aesthetic preferences are thus moderated by notions of autonomy that are, in turn, enhanced by expectations of self-efficacy.

The role of social forces in the dynamics of aesthetic appreciation is not without controversy. In *The Clockwork Muse* Martindale (1990) made a strong case that artistic change is stifled rather than inspired by social influences. Instead, he pointed out that some intrinsic pressure for novelty and distinction shapes individual artistic careers and trends. According to Martindale, this balance wheel works against “social forces [which] are analogous to friction, in that they impede or slow down the progress of an artistic tradition” (pp. 34–35). At first glance, this may sound contradictory to the approach advocated in the present article. But in fact, Martindale’s remarks nicely summarize an essential aspect of our model by assuming a perpetual conflict between needs for autonomy and affiliation on an individual level and between innovation and tradition on a cultural level. Nevertheless, drawing on our model, we dispute Martindale’s claim that artistic change would flourish in a “social vacuum” (p. 34): Outside of a social context, there is apparently no need to strive for autonomy and distinction. For a person in the position of Robinson Crusoe our model predicts a strong desire for safety and affiliation.^11^ Under such conditions, we expect Mr. or Mrs. Crusoe to indulge in keepsakes and souvenirs, rather than to contemplate on challenging artworks. Apparently, Martindale’s view on aesthetics is strongly influenced by a modernist concept of art emphasizing change and novelty. His model sympathizes with the autonomous artist who bravely struggles against an oppressive tradition in his/her quest for a unique point of view and a distinctive artistic signature. Due to the extraordinary efforts of exceptional individuals “the progress of an artistic tradition” (p. 34) is maintained. With this statement Martindale clearly refers to the “antitraditional tradition” (Cǎlinescu, 1987, p. 66) of Modernism. We make a case that a modernist view has placed its mark on the most influential theories of empirical aesthetics (Ortlieb and Carbon, 2018). For instance, in *Aesthetics and Psychobiology* Berlyne (1971) stated that collative stimulus properties such as novelty, surprise, and ambiguity form the “essential ingredients of art and of whatever else is aesthetically appealing” (p. viii). Apparently, both Martindale and Berlyne committed themselves to an “aesthetic of deviation” (Fricke, 2000), which is based on the premise, that whatever is aesthetically pleasing has to stand out from the beholder’s previous experience questioning his or her normative expectations. This approach to aesthetics certainly has its place. Yet, unfortunately, it has narrowed our view to the perspective of the self-contained individual in the pursuit of promotion goals and autonomy. As a consequence, the importance of arousal for aesthetic pleasure has been overrated in Western art-related theories (e.g., Berlyne, 1971), whereas the role of familiarity in aesthetics for maintaining a cultural worldview, social identity formation, and group cohesion has been neglected (see Dissanayake, 1990). The affirmative function of aesthetics and its social dimension has received little attention in empirical aesthetics. Under the influence of a “modernist” approach to aesthetics, it seems that we have lost sight of some highly relevant aesthetic phenomena, including the entire field of popular aesthetics (e.g., kitsch, folk art, religious rites, and customs). Interestingly, a complementary view upon the arts can be found among scholars from anthropology, design theory, and social psychology. For instance, Dissanayake (1990) claimed that art production evolved from a collective effort to deal with uncertainties of nature by symbolically exerting control over it. According to Dissanayake, art attempts to make particular things, locations, events, and behaviors *special*—i.e., more salient, pleasurable, and therefore memorable—because they are important either for survival or social cohesion. By turning artifacts into devotional objects, locations into sacred places, and behaviors into rituals or customs, premodern artists made certain objects and events stand out from everyday experience. Yet instead of thwarting the beholder’s normative expectations, these practices help to establish and maintain a common worldview. Today, it seems that this affirmative function of art still applies to various phenomena of popular culture, particularly kitsch. In his book on *Emotional Design* Norman (2004) claimed that souvenirs and keepsakes are special to us because they refer to friends and relatives thereby evoking pleasant memories of important episodes of our life. By instilling us with feelings of communality and affection these artifacts may cheer us up on a rainy day (Norman, 2004). Based on Kulka’s (1996) definition, we have made a case that these reassuring qualities are ideally represented by kitsch. In empirical aesthetics the discovery of “attitudinal effects of mere exposure” by Zajonc (1968) has inspired extensive research on the hedonic value of processing fluency and raised awareness for the important role of familiarity in aesthetic appreciation (Reber et al., 2004). In social psychology, this turn away from collative variables has led to a strikingly different understanding of art. *Terror Management Theory* (TMT), for example, states that the main psychological function of art is to provide “opportunities to bolster cultural belief systems that provide death-transcending meaning and significance” (Landau et al., 2010, p. 114). With reference to Dissanayake’s (1990) ideas, TMT highlights the reassuring function of art in “social life, including rituals to ensure success in group ventures, rites of passage, recognition of seasonal changes, and memorial occasions” (Landau et al., 2010, p. 115). Consequently, the terror management approach to aesthetics adopts a definition of art—“culturally significant meaning, skillfully encoded in an affecting, sensuous medium” (Anderson, 1990, p. 238, quoted from Landau et al., 2010)—which is perfectly in line with Kulka’s (1996) kitsch criteria, but seems hardly compatible with the emancipatory aims of avant-garde art (e.g., Dadaism).^12^ In the case of TMT, scholars are primarily interested in the effects of existential threats on aesthetic judgments. Hence, unlike theories proposed by Martindale and Berlyne, TMT puts special emphasis on the alienated individual (i.e., unsettled by mortality concerns) and its needs for relatedness and meaning. By looking at aesthetic phenomena under a prevention focus, it is not surprising that priority is given to the reassuring aspects of art. A considerable body of research shows that reminders of mortality amplify people’s—positive and negative—aesthetic judgments (Landau et al., 2010): Artworks that appear accessible based on the beholder’s cultural worldview are rated more positively, whereas artworks which defy meaningful interpretation are rated more negatively. These findings are consistent with the basic assumption of our model that people prefer aesthetic stimuli that allow for immediate apprehension, whenever they feel vulnerable and dependent.

What is art for? Answers to this question obviously turn out quite differently depending on the theoretical vantage point: Looking at the self-contained individual, scholars tend to emphasize an intrinsic appetence for change and novelty and an emancipatory function of art (e.g., Berlyne, 1971; Martindale, 1990; Nietzsche, 1882/2001). By contrast, when research is focused on the vulnerable individual, special emphasis is placed on needs for safety and relatedness. As a result, the reassuring function of art is put forward. Clearly, each of these approaches to dynamics in aesthetic appreciation has its merits. However, both appear one-sided as they presuppose different motivational states (i.e., different regulatory foci) in the observer, respectively the artist. According to our model, these perspectives are not mutually exclusive. In fact, they represent two sides of the same coin: A psychobiological mechanism which has evolved to balance needs for autonomy and intimacy in social living animals. We claim that changes in aesthetic appreciation can only be understood if we discriminate between two types of aesthetic experience—a fluent and a disfluent one—which account for these conflicting needs. Moreover, we argue that the state of tension between them is ideally represented by the polemic opposition of kitsch and art. Hence, the dynamics which result from a perpetual conflict between autonomy and relatedness modulate our motivation to engage with the one or the other. Such dynamics are also discernible on a cultural level regarding the relationship between kitsch and art: A recent study by Hanquinet et al. (2014) suggests that Bourdieu’s dichotomy of popular (based on beauty and harmony) and highbrow aesthetics still plays an important role, although “the content of highbrow aesthetics has changed, now privileging ‘postmodernist’ dimensions over modernist ones” (p. 111). This change of common taste has brought about a fundamental reevaluation of kitsch: Since the late 1960s, Pop Art and Postmodernism have blurred the distinction of kitsch and avant-garde art. Today, paintings and sculptures featuring prototypical attributes of kitsch are recognized as high art (e.g., Jeff Koons). Can our model account for this shift in aesthetic evaluation? In an interview the former director of Tate Modern, Chris Dercon, stated that contemporary art no longer intends to separate, shock or polarize, but to provide guidance and relatedness: “[I]n a world that is becoming more and more complex, which nobody can overview, people strive for a sense of belonging” (Sebastian and Dercon, 2014, p. 1, translation by the authors) and he predicted that before long “we will be searching for artworks which help us to remember. The old will become more important than the new” (p. 2, translation by the authors). Given Dercon’s premise, that the world is becoming more and more confusing, our model makes precisely the same prediction: In the face of increasing uncertainty, we expect people to seek for the “warm glow of familiarity” across different domains, including art.^13^

## Conclusion, Explanatory Scope, and Future Research

So far we have made a case that seemingly contradictory findings and theoretical concepts from various disciplines may be reconciled, if they are related to mechanisms of social distance regulation: By drawing on the *Zurich Model of Social Motivation* (Bischof, 1975, 1993, 2001) and *Regulatory Focus Theory* (Higgins, 1998) we are able to specify conditions under which novel stimuli should become more attractive than familiar ones and vice versa. In general, our model predicts that whatever affects people’s notion of security and self-efficacy will yield in a shift of regulatory focus and thus affect aesthetic judgments. Finally, we would like to make some closing remarks on the explanatory scope of our model and to point out possible limitations as well as implications for future research on dynamics of aesthetic appreciation.

First and foremost, our model suggests a dynamic interrelation between aesthetic evaluation and critical life events. For instance, any episode in life during which needs for security and attachment are significantly augmented (e.g., couples expecting a baby) should be associated with a greater susceptibility for familiar, easy-to-process-stimuli, whereas life events that boost people’s sense of achievement (e.g., passing one’s final exams) and autonomy (e.g., leaving the parental home) should coincide with a state of increased appetence for novelty also in the aesthetic domain. Resting on the Zurich Model with its psychobiological foundations, our model can account for developmental aspects such as a characteristic shift in aesthetic taste during adolescence. Due to a general inhibition of the autonomy system throughout infancy and a sharp increase of its reference variable (autonomy claim) with the onset of puberty, we expect appetence for extremely arousing imagery to peak during adolescence. Market research on target groups of horror films supports this assumption (see Blothner, 2004).^14^ Yet, based on our model we would also expect to find a particularly high reluctance to familiar stimuli reflecting parental taste. In the context of inter-generational conflict, the term kitsch is commonly used to ridicule the outdated aesthetic standards of an older generation (Avenarius, 1920; Stemmle, 1931; Dettmar and Küpper, 2007).

Social motivation is one of the broad themes in research on gender differences (Eagly and Wood, 1991; Feingold, 1994). In a cross-cultural study including participants from 26 cultures by Costa et al. (2001), women scored higher than men in warmth and gregariousness, but lower in assertiveness and excitement seeking. With reference to findings from child development, Bischof-Köhler (2006) suggested that needs for safety and relatedness, respectively arousal and autonomy, are weighted differently in males and females: If this is the case, aesthetic preferences should differ accordingly. For instance, we would expect males and females to respond differently to artworks with troubling content (Chamorro-Prezumic et al., 2010; Ortlieb et al., 2016b) or certain film genres (horror films; Blothner, 2004). In an experimental study by Wühr and Schwarz (2016) men recalled details from action films more accurately than women, while women excelled in recalling content from romantic films. Interestingly, this gender gap is not a stable phenomenon: A survey on film preferences in older adults (aged 50+) showed that “[w]ith increasing age, older men prefer film genres that otherwise tend to be preferred by female viewers. Women, as they are older, tend to increasingly prefer female film content” (Hoffmann and Schwender, 2007, p. 473). When Csikszentmihalyi and Rochberg-Halton (1981) interviewed members of 82 US-families about their most cherished possessions, a similar pattern emerged: In the parent’s generation, men mentioned objects which were associated with action and/or personal achievements (e.g., tools and trophies) more often than women, who in turn put more emphasis on objects reflecting interpersonal relationships and/or nurturant behavior (e.g., heirloom and plants). However, as in the case of film preferences, these gender differences were leveled out among older respondents: In the grandparent’s generation both men and women stressed objects referring to memories and self-transcendence (e.g., photos). It would certainly be worthwhile to examine whether such long-term dynamics in gender-related aesthetic preferences reflect changes in social motivation.

Kitsch is a truly international term (see Ortlieb et al., 2017). However, any culture (or subculture) will place its stamp on the innate mechanism of social motivation by putting special emphasis on certain needs (e.g., autonomy) and by devaluing others (Dissanayake, 2015). Thus, a cross-cultural comparison of kitsch concepts between individualistic and more collectivistic societies could be enlightening. According to Hofstede (2001), members of highly individualistic cultures tend to prefer autonomy over strong social ties. They are expected to take care of themselves (e.g., self-made man/woman) and encouraged to swim against the mainstream. By contrast, collectivistic societies place special emphasis on a common worldview and strong social bonds. Consequently, members of collectivistic societies are expected to tune down their autonomy claim in exchange for security and relatedness. Clearly, cross-cultural differences in terms of collectivism and individualism touch upon the basic variables of our model. In popular culture of modern individualistic societies Dissanayake (2015) observed an obsession with harmony and romantic love that have been barred from high art. By contrast, totalitarian states can be seen as corner cases (or grotesque caricatures) of collectivistic societies. Its individual members submerge in a whole, which is exclusively dedicated to serving a higher idea. An abstract common good is given absolute priority over any individual desire for autonomy which is officially devalued and violently suppressed (e.g., Nazi-propaganda slogan “You are nothing, your people is everything”). Analyses of right- and leftwing totalitarian societies suggest that under the influence of any collectivistic ideology artistic production will inevitably yield in kitsch production (Greenberg, 1939; Kundera, 1984/1999; Friedländer, 1985/2007).

Apart from basic research on dynamics in aesthetic appreciation, we are convinced that our model can inform applied research. Social motivation is about dynamics of attachment and detachment. Thus, our model should be of practical use for product design (e.g., design evaluation) and marketing (e.g., measures to establish and maintain consumer ties). In terms of design evaluation, we can specify conditions under which innovation is likely to be appreciated: For instance, we recommend providing an environment in which people feel safe and competent, if we wish for them to gratify futuristic design (Carbon et al., 2013). By contrast, we expect uncertain contexts (e.g., traveling a foreign country) to increase the “glow of warmth” radiated by traditional products^15^ or familiar brands (e.g., Coca Cola^TM^ or Starbucks^TM^).

Certainly, our model has its limitations: Based on a systems theoretical approach to social motivation which was originally devised from an evolutionary, ethological, and developmental perspective, it is only partially compatible with trait constructs from personality research that figure prominently in empirical aesthetics (e.g., Extraversion, Openness, Schizotypy, etc.). This makes it difficult to relate certain findings on aesthetic preferences and personality to the model we have outlined. Schönbrodt et al. (2009) have developed a set of standardized scales for the assessment of interindividual differences in security, arousal, power, prestige, and achievement, that is explicitly based on the Zurich Model. In fact, content-validity of the *Motive Profile following the Zurich Model* (MPZM) has been cross-checked and approved by the author of the Zurich Model (Schönbrodt et al., 2009). When construct validity of the MPZM was studied in a multitrait-multimethod analysis using the German adaptation of the *NEO-Five-Factor Inventory* (NEO-FFI; Körner et al., 2002), the *Multi-Motive Grid* (MMG; Schmalt et al., 2000), and the *Personality Research Form* (PR-D; Stumpf et al., 1985) it only “showed convergent validity to content-matched scales of the PR-D (*r* = 0.55), [but] no differentiated relationship to the MMG, and few correlations to the NEO-FFI” (p. 141). In a study on external validity including biographical data, however, the MPZM outperformed both the NEO-FFI and the MMG in terms of predictive power and incremental validity (Schönbrodt et al., 2009). Albeit our theoretical framework is not well-aligned with standard models of personality research, there is first indication that the MPZM, the anxiety-related coping inventory (ABI) by Krohne and Egloff (1999), and the *General Self-Efficacy Scale* (GSE) by Schwarzer and Jerusalem (1995) make appropriate measures for the empirical study of the model’s main propositions (Muth et al., 2017b; Fischer et al., 2018; Vlasova et al., 2018).

## Author Contributions

SO had the initial idea to bring the Zurich Model together with dynamics of aesthetic appreciation, mainly wrote the manuscript, and created the figures. C-CC brought insight from empirical aesthetics. SO and C-CC worked further on the manuscript and finished it together.

## Conflict of Interest Statement

The authors declare that the research was conducted in the absence of any commercial or financial relationships that could be construed as a potential conflict of interest.

## Footnotes

1 In France and England for instance, particular styles are identified with the reigns of certain monarchs (e.g., “Louis-XVI,” “Empire,” “Georgian,” etc.). With the breakdown of monarchies in Russia and Germany, revolutionary art movements (e.g., Cubo-Futurism, Suprematism, Dadaism) flourished before they were silenced by totalitarian art doctrines in the early 1930s.

2 Meanwhile, the term “avant-garde” is used as a label for an epoch in art history. In the following, it refers to a socio-critical concept of art, which is not limited to a certain period.

3 In this article the following distinction between “security” and “safety” is made: While safety merely indicates the absence of potentially harmful stimuli, security refers to the unique combination of intimacy and safety that is conveyed by familiar and trustworthy others.

4 *Regulatory Focus Theory* by Higgins (1998) proposes a basic distinction between two motivational orientations: A *prevention focus* that motivates us to anticipate and avoid potential threats and a *promotion focus* that draws our attention towards opportunities for growth and achievement.

5 For a comprehensive overview of etymological hypotheses see Cǎlinescu (1987).

6 The present article is mainly concerned with the psychological effects of kitsch. For a more detailed analysis of kitsch-related stimulus properties see Ortlieb and Carbon (2018).

7 Although Berlyne (1971) spoke of object properties, *collative variables* clearly lie in the eye of the beholder as judgments of novelty and complexity will always depend on one’s previous experience. Only in the rare case of truly groundbreaking artworks (e.g., Picasso’s *Desmoiselles d’Avignon*), a painting or a sculpture may be ‘objectively’ innovative with regard to the entire art world.

8 Recurrent nightmares and intrusive images in patients with post-traumatic stress disorder cast serious doubt on a general fluency-positivity link. Alternatively, the *Fluency Amplification Model* by Albrecht and Carbon (2014) postulates that processing fluency does not necessarily amplify positivity of a stimulus, but the intensity of its initial valence. According to Albrecht and Carbon, the fluency-positivity link only applies to stimuli with a positive valence; in the case of aversive stimuli, fluency increases negativity. Regarding kitsch, this particular limitation of the HFM is not an issue, since kitsch classification presupposes content with a positive valence (Ortlieb and Carbon, 2018).

9 As Csikszentmihalyi and Rochberg-Halton (1981) pointed out “the words familiar and family are both derived from the Latin word *familia*, which included the kin as well as household possessions” (p. 185).

10 According to Dettmar and Küpper (2007), the Kantian opposition of *free* and *dependent* beauty can be seen as one of the precursor concepts of the dichotomy of art and kitsch.

11 In the movie “Cast away” which is loosely based on Defoe’s novel, the only survivor of an airplane crash creates a fictitious counterpart: In desperate need for affiliation, he paints a human face onto a volleyball and maintains a social relationship with “Mr. Wilson.”

12 The aim of Dadaists was to ridicule and undermine the dominant worldview of their contemporaries, not to skillfully codify “culturally significant meaning” (Anderson, 1990, p. 238, quoted from Landau et al., 2010).

13 Interestingly, contemporaneous trend analysts spoke of a “New Generation Biedermeier” (Krüger, 2014, translation by the authors) as an increasing number of young people valued safety and intimacy over autonomy and achievement. According to Grünewald (2013), this withdrawal from the public into the private sphere reflects a general tendency to escape from the growing demands of meritocracy in a globalized world.

14 Blothner’s (2004) analysis of film consumption in Germany showed that preference for horror films was highest among (male) teenagers between 15 and 19 years. However, this fascination with horror films was reduced among participants in their early twenties. In all other age groups older than 25 years, the horror genre played only a marginal role.

15 The city of Bamberg is located in Upper Franconia, the region with the highest density of small, privately owned breweries in the world. From their personal experience the authors of this paper report warm feelings of belonging, whenever they encounter a traditional beer from these premises elsewhere.
